# Utilization of acute and long-term care in the last year of life: comparison with survivors in a population-based study

**DOI:** 10.1186/1472-6963-9-139

**Published:** 2009-08-05

**Authors:** Anne Margriet Pot, France Portrait, Geraldine Visser, Martine Puts, Marjolein I Broese van Groenou, Dorly JH Deeg

**Affiliations:** 1 EMGO Institute for Health and Care Research, VU University Medical Centre, Amsterdam, the Netherlands; 2 Department of Clinical Psychology, Faculty of Psychological and Educational Sciences, VU University, Amsterdam,the Netherlands; 3 Institute of Health Sciences, Faculty of Earth and Life Sciences, VU University, Amsterdam, the Netherlands; 4 Department of Sociology, Faculty of Social Sciences, VU University, Amsterdam, the Netherlands; 5 Solidage Research Group, Centre for Clinical Epidemiology and Community Studies, Jewish General Hospital, McGill University, Montreal, Canada; 6 Department of Psychiatry, VU University Medical Centre, Amsterdam, the Netherlands; 7 Netherlands Institute of Mental Health and Addiction, Utrecht, the Netherlands

## Abstract

**Background:**

It is well-known that the use of care services is most intensive in the last phase of life. However, so far only a few determinants of end-of-life care utilization are known. The aims of this study were to describe the utilization of acute and long-term care among older adults in their last year of life as compared to those not in their last year of life, and to examine which of a broad range of determinants can account for observed differences in care utilization.

**Methods:**

Data were used from the Longitudinal Aging Study Amsterdam (LASA). In a random, age and sex stratified population-based cohort of 3107 persons aged 55 – 85 years at baseline and representative of the Netherlands, follow-up cycles took place at 3, 6 and 9 years. Those who died within one year directly after a cycle were defined as the "end-of-life group" (n = 262), and those who survived at least three years after a cycle were defined as the "survivors". Utilization of acute and long-term care services, including professional and informal care, were recorded at each cycle, as well as a broad range of health-related and psychosocial variables.

**Results:**

The end-of-life group used more care than the survivors. In the younger-old this difference was most pronounced for acute care, and in the older-old, for long-term care. Use of both acute and long-term home care in the last year of life was fully accounted for by health problems. Use of institutional care at the end of life was partly accounted for by health problems, but was not fully explained by the determinants included.

**Conclusion:**

This study shows that severity of health problems are decisive in the explanation of the increase in use of care services towards the end-of-life. This information is essential for an appropriate allocation of professional health care to the benefit of older persons themselves and their informal caregivers.

## Background

Care utilization in the last year of life of older adults is an important, but still relatively unexplored field. Research in this area has been conducted primarily from an economical and political point of view, focusing on its costs [1-5]. These studies have shown that at any age, the last year of life is more costly in terms of care utilization than any earlier year of life. In addition to costs, knowledge of the types and determinants of health care utilization in the last phase of life is important. Health conditions and concomitant health care utilization at the end of life are often complex, and clarifying the types and determinants of health care utilization may facilitate the appropriate allocation and organization of health care for those of whom we think are in their last phase of life. [6]. This in turn will be of benefit for older people themselves and their informal caregivers, for whom the psychological, physical, social and financial impact of the last year of life may be tremendous [7].

In recent years, retrospective studies from several countries reported on the place where people die, showing that a substantial proportion died in hospital, with percentages ranging from around 45% to 60% [8-13]. Even a significant proportion of those who live in a nursing home may end their lives in hospital (e.g. 29% in South-West Germany [14]). However, empirical evidence on other types and determinants of care utilization in the last phase of life is still scarce.

A frequently used framework of determinants of health care utilization, developed by Andersen and Newman [15], distinguishes three domains: 1) personal attributes that *predispose *individuals to seek care (e.g., age, gender); 2) factors that *enable *access to care (e.g., income, having a partner); and 3) factors that reflect the *need *of care (e.g., disease, disability). Studies on care utilization in the last year of life show the importance of these three domains of determinants [9,16,17]. These studies also show the importance of distinguishing different types of acute and long-term care, because determinants varied greatly across types. Bickel [9] showed that in the last year of life the use of professional home care was predicted by living alone and especially by the need of care. Hospital admission was predicted by younger age, whereas the use of long-term care facilities was predicted by older age, being widowed or never married, having children, and especially by the need of care. Jakobsson et al. [17] showed that in the last three months of life hospital admission was predicted by living alone and not having dementia, whereas hospital-based outpatient care was predicted by living with other(s) and having dementia. The use of long-term care facilities was predicted by older age and especially living alone and being ADL-dependent, and care in private homes was predicted by having neoplasms or musculoskeletal disease(s), and not having dementia. Although the studies cited covered a variety of care services, no attention was paid to informal care.

Previous studies used samples of older adults who died in a specific time period, and did not provide comparative information on care utilization in persons who were not in their last phase of life. Nevertheless, this comparison is essential to evaluate properly the types and determinants of care utilization in the last phase of life.

The population-based Longitudinal Aging Study Amsterdam (LASA) covers information on a wide range of health-related and psychosocial factors as well as on acute and long-term care utilization, including professional and informal care. The information gathered includes predisposing, enabling and need factors. Based on these data, we studied the utilization of acute and long-term care among older adults in their final year of life as compared to those who survived over at least three years. Secondly, we examined whether the differences in utilization between older adults in their last year of life and older adults not in their last year of life could be accounted for by predisposing, enabling or need characteristics of the older adults.

## Methods

### Sample

The current study is part of the Longitudinal Aging Study Amsterdam (LASA), an ongoing multidisciplinary study on predictors and consequences of changes in well-being and autonomy in the older population [18]. The sampling and procedures adopted to achieve the baseline sample and the response rates at baseline and follow-up have been described in detail in previous publications [19]. What follows here is a summary of the main design characteristics.

At baseline, the random, nationally representative, age and sex stratified sample consisted of 3107 older adults (55 – 85 years). The sample was drawn from the population registries of 11 municipalities in three regions of the Netherlands: the west, the north-east, and the south. Data were gathered in face to face interviews at baseline (1992–93) and in subsequent cycles three years apart. Respondents were interviewed in their homes by specially trained and intensively supervised interviewers. Informed consent was obtained prior to the study, in accordance with legal requirements in the Netherlands. The research was carried out in compliance with the Helsinki declaration, and was approved by the ethics committee of the VU University Medical Centre.

The present study uses data from the first three cycles of LASA and the 1-year and 3-year mortality information following each cycle, i.e. the period 1992–2002. Information on mortality was obtained from the municipalities in which the respondents were living at their time of death. Two groups were distinguished: 1. Survivors: respondents who were still alive at the next cycle, and; 2. End-of-life group: respondents who died within 12 months after a cycle. Excluded were respondents who died in the second or the third year after one of the cycles. We focused on all respondents who died within one year after a cycle, and those who were still alive three years after a cycle, to ensure a meaningful contrast between both groups, based on the acceleration of health care utilization in the last year of life. This approach is common in end-of-life care studies [e.g. [20]]. If we had included also those who had died one to three years after the cycles, we would have had no recent data of these respondents. Recent data are important, because there might be many changes in the last year of life. The numbers of respondents excluded due to death between one and three years of follow-up were 252 at cycle I, 227 at cycle II, and 231 at cycle III.

In addition, insufficient information was available on respondents who refused or were too frail to participate in cycle II or III (145 and 139 respondents, respectively) and on respondents with a telephone interview at cycle II or III (186 and 175 respondents, respectively). Therefore, these respondents were excluded as well.

The final numbers of respondents included are 2,855, 2,108, and 1,703 at cycles I, II, and III, respectively (Table 1). Note that in the present study sample, respondents were included one, two, or three times according to their survival status. Thus, survivors at cycle III who participated in cycles I, II, and III were included three times, whereas others were included one or two times only. We adjusted for this in the analyses, by using Generalized Estimated Equations (GEE: see Analyses).

**Table 1:** Composition of study sample

	Alive 3 years after cycle	Deceased 1 year After cycle	Total
Cycle I	2,738	117	2,855
Cycle II	2,028	80	2,108
Cycle III	1,638	65	1,703

Total	6,403	262	6,665

In order to examine the effect of exclusion due to missing data, the present study sample at cycle II, excluding drop-outs and those with telephone interviews, was compared with the total sample at cycle II. The second wave was the first in which telephone and proxy interviews were used if a respondent refused a face-to-face interview. The percentage of deaths within one year after cycle II was 3.8% in the study sample and 3.9% in the total sample. Additional analyses showed that second wave respondents who had a telephone interview were not more likely to die within three years than face-to-face respondents. Second wave respondents for whom no or proxy data were available were three times more likely to die within three years (OR = 3.1). However, this excess mortality risk was reduced to insignificance (OR = 1.5) when taking into account age and functional limitations at wave 1. Because all our models take into account age and functional limitations, the attrition due to incomplete data is not likely to influence our results.

### Measures

#### Outcomes

#### Acute and long-term care

*Acute care *services included contact with medical specialist(s) and hospital admission in the past six months, each coded as 0 = no and 1 = yes. *Long-term care services *included current receipt of informal or professional home care and institutional care. Informal and professional home care was restricted to personal care (such as dressing and bathing) and excluded housekeeping assistance, as personal care is more pertinent to end-of-life care. *Informal care*, provided by the partner, children, other family members, neighbors or acquaintances, was coded as 0 = no informal care, and 1 = informal care. *Professional home care*, provided by a home care service, was coded as 0 = no professional home care, and 1 = professional home care. Professional home care implies government subsidized care. Only one person in the study sample paid for personal care out-of-the-pocket. Finally, *institutional care*, provided to those living in a residential or nursing home or a psychiatric hospital, was coded 0 = community living, 1 = institutionalized.

### Determinants

*End-of-life status *was coded as 0 = survived longer than three years directly following a cycle, and 1 = died within one year directly following a cycle.

*Predisposing characteristics *were represented by age (in years) and gender (1 = male, 2 = female).

*Enabling characteristics *included were: level of education (low, middle, high), monthly income in 1000 euro, urbanicity (low, middle, high), partner status (0 = no partner, 1 = partner), and number of children living in the neighborhood (travel-time less than 15 minutes).

A broad spectrum of *need characteristics w*as included. *Physical functioning *was measured both by self-reports and by performance tests of physical ability. *Self-reported *functional limitations were assessed based on three items (e.g. "Can you walk up and down 15 steps?"), with five response categories each: 0 = able without difficulty, 1 = able with some difficulty, 2 = able with much difficulty, 3 = only with help, 4 = not able. The scores were summed to a scale ranging from 0 to 12 [21,22]. The *performance tests *measured the number of seconds needed to complete three tasks, for example to walk three meters back and forth along a line. The quartiles of the time needed were coded as 1 = fast, through 4 = slow. Those who could not do the test were assigned the code 5. The scores were summed to a scale ranging from 3 to 15 [23,24]. *Disability *was assessed by asking respondents whether health problems limited their daily activities using a 3-point scale (0 = no, 1= mildly or moderately, 2 = severely). The presence of *chronic diseases *was assessed by asking the participants whether they had any of the following diseases: chronic obstructive pulmonary diseases (COPD), heart diseases, peripheral artery diseases, stroke, diabetes, arthritis, or cancer. The number of chronic diseases was calculated to indicate multimorbidity [25]. The Mini Mental State Examination (MMSE) was used to assess *cognitive status *[26]. The score ranges between 0 and 30 and a score of 23 or below is used to indicate cognitive impairment [27]. The Center for Epidemiological Studies Depression Scale (CES-D) was used to measure *depressive symptoms *[28,29]. The scores range from 0 to 60. A score of 16 and higher is interpreted as indicative of clinically relevant depression. *Perception *was measured using self-report items on difficulty seeing and hearing [22]. *Self-perceived health *was measured using one item, with codes from 1 = very good, to 5 = poor [30].

### Analyses

We examined the differences between the characteristics of the survivors and the deceased at each cycle. For continuous variables we used t-tests for independent groups at each cycle and for categorical variables chi squared tests for each cycle. Care utilization was explored by comparing respondents in their last year of life with survivors. Determinants of care utilization were examined using logistic regression models based on generalized estimating equations (GEE) [31,32]. GEE-analysis can be seen as a regression analysis which takes into account that the same subjects are measured over time implying that their measurement values are not independent. Moreover, GEE retains subjects with data on one or two waves. In GEE, the standard errors of the estimates are corrected for repeated measurement. An exchangeable correlation matrix was assumed.

Five models were analyzed, each with one use of care variable as the dependent variable, end-of-life status as the main determinant, and predisposing, enabling and need characteristics as potential explanatory variables. For each model, three analyses were carried out. In a first analysis, regression coefficients were estimated for the relationship between end-of-life status and the outcome variables 'use of care' at time t_x _(x = 1, 2 or 3), adjusting for the predisposing variables at t_x_. In a second and third analysis, regression coefficients were estimated for the relationship between end-of-life status and the outcome variables at t_x _(x = 1, 2 or 3), additionally adjusting for enabling and need variables at t_x_. In all analyses, the model was adjusted for time of measurement of the outcome variables as a continuous variable, because data were included from three different cycles. A preliminary test showed that the interaction 'time of measurement × group status' was not significant. Thus, the differences in use of care services between the end-of-life group and the survivors did not change significantly across waves. This means that we can pool the results across the waves. In another set of preliminary analyses, all variables used as continuous in the models were checked for linearity of association by defining them as categorical.

## Results

### Characteristics of end-of-life and survivor groups

The size of the end-of-life group was observed to be 4.1% at cycle I, 3.8% at cycle II and 3.8% at cycle III (Table 1).

Table 2 shows the characteristics of the survivors and the end-of-life group at cycles I, II and III. The use of all types of care, but especially the use of long-term care, was higher among the end-of-life group as compared to the survivors (Table 2, top section). Whereas substantial numbers of survivors had contacts with medical specialists or were admitted to a hospital during the past six months (e.g. at cycle II, 47% and 9%, respectively), only few of the survivors used long-term care (ranging from 1.9% to 3.4%, depending on type of care and cycle). In contrast, the majority of the end-of-life group had contacts with a medical specialist, and on average 20% were admitted to a hospital, 25% used professional home care, and 20% lived in a care institution. Compared to the other types of care, informal personal care was received relatively little (on average 10% of the end-of-life group).

**Table 2:** Characteristics of survivors and end-of-life group at each cycle: use of care, predisposing and enabling factors

	Cycle I Survivors (n = 2,737)	Deceased (n = 117)	Cycle II Survivors (n = 2,028)	Deceased (n = 80)	Cycle III Survivors (n = 1,638)	Deceased (n = 65)
***Use of care indicators *(%)**						
Acute care: Contact with medical specialists	44.9	61.6*	47.0	57.3*	48.9	50.8*
Acute care: Hospital care	9.5	24.2**	9.3	19.5**	8.2	16.4**
Long-term care: Informal personal care	1.9	11.1***	1.3	7.5***	1.9	10.8***
Long-term care: Professional home care	1.9	20.5***	2.6	27.5***	3.2	24.6***
Long-term care: Institutional care	2.4	17.1***	2.7	22.5***	3.4	18.6***

***Predisposing variables***						
Age (mean)	69.9	77.0***	71.7	79.0***	73.2	83.2***
Female (%)	53.5	36.8**	55.3	40.0**	56.8	39.0**

***Enabling variables***						
Education (%)						
Low	62.5	69.9	60.1	71.3	59.0	57.6
Middle	25.7	18.6	27.7	13.8	28.6	23.7
High	11.7	11.5	12.2	15.0	12.4	18.6
Income in Euros (mean)	1,135	1,041	1,180	1,091	1,193	1,221
Urbanicity (%)						
Low	24.5	17.1	25.0	25.0	25.9	15.3
Middle	24.3	26.5	24.2	20.0	24.4	25.4
High	51.2	56.4	50.8	55.0	49.8	59.3
Partner (%)	67.7	53.0**	63.9	51.3**	62.1	47.5**
Children nearby (%)	62.0	57.0	61.0	46.0	61.2	63.3

Significance tests at each cycle: * significant at p < 0.05, ** significant at p < 0.01, *** significant at p < 0.001

**Figure 1:**
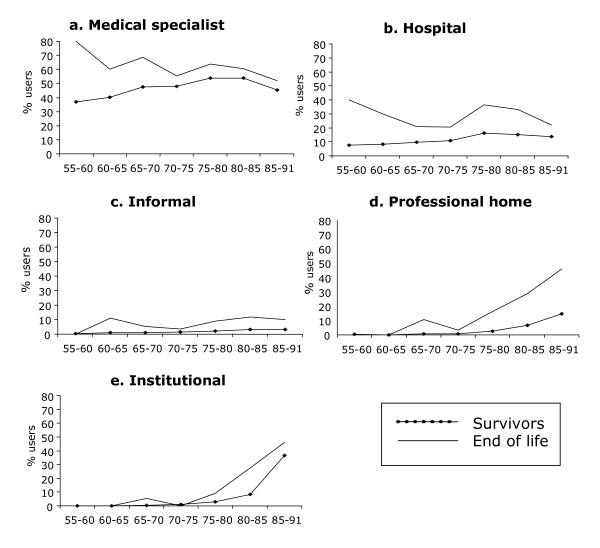
**Percentage users by age for each end-of-life status, all three cycles taken together**. a. Medical specialist, b. Hospital care, c. Informal care, d. Professional home care, e. Institutional care

The end-of-life group was on average about 8 years older and consisted of more males than the survivors, a pattern that persisted across cycles (Table 2, second section). Figure 1a–e show the age pattern of the use of care among older adults in their final year of life as compared to those who survived over at least three years. First, the use of acute care (hospital admission and contacts with medical specialists) by survivors increased linearly with age until about 80 years and then stabilized, whereas in the end-of-life group, the use of acute care decreased with age from age 80. In the use of professional long-term care (home care and institutional care), an increase with age was found for both groups. Informal care did not show a clear age trend. However, the numbers at younger ages are small and should be interpreted with caution. Second, at all ages the end-of-life group used all types of care to a greater extent than the survivor group. Notably, a different age pattern was found for long-term and acute care. At younger ages, the difference between the end-of-life group and the survivor group was most pronounced for the use of acute care, whereas at older ages it was most pronounced for the use of long-term care. The discrepancy between the end-of-life group and the survivor group was largest for admission to a hospital. For instance at age 62.5, the end-of-life group was 1.5 times more likely to have contact with a medical specialist, and was 4 times more likely to be admitted to a hospital than survivors. With respect to long-term care, the discrepancy between the end-of-life group and the survivor group was relatively small at younger ages and increased with age. The discrepancy between both groups was largest for professional home care. For instance at age 77.5, the end-of-life group was 6 times more likely to be using professional home care, 4 times more likely to be using informal care, and 3 times more likely to live in a long-term care facility as compared to the survivor group.

With respect to the enabling characteristics, respondents in the end-of-life group lived more often without a partner as compared to respondents who survived (Table 2, third section). The end-of-life group did not differ significantly from the survivors with respect to the other enabling characteristics.

Regarding the need characteristics (Table 3), the end-of-life group was more disabled, had more functional impairments and a poorer performance as compared to the survivors. They had a higher number of chronic diseases (on average 2.2 versus 1.6). They also showed a higher prevalence of all diseases except for arthritis. Furthermore, they were more likely to have vision impairment (about 32% vs 22%), scored substantially higher on cognitive impairment (about 27% versus 6%), were twice as likely to have clinically relevant depressive symptoms (about 24% versus 12%) and to perceive their health as fair to poor (about 58% versus 35%) as compared to the survivors. The difference in hearing impairment did not reach statistical significance. Most of the differences observed persisted across the three cycles. However, at cycle III, the differences between the end-of-life group and the survivors with respect to diabetes and cancer were not significant.

**Table 3:** Characteristics of survivors and end-of-life group: need factors

	Cycle I Survivors (n = 2,737)	Deceased (n = 117)	Cycle II Survivors (n = 2,028)	Deceased (n = 80)	Cycle II Survivors (n = 1,638)	Deceased (n = 65)
***Need variables***						
Functional limitations (%)						
Score ≥ 2	26.1	62.2***	32.6	68.8***	35.9	78.6***
Physical performance (%)						
Score 3–5	31.3	8.3	33.0	15.4	27.2	8.5
Score 6–7	26.5	18.8	24.2	13.8	22.9	10.6
Score 8–10	26.7	34.4	27.4	27.7	30.1	27.7
Score 11–15	15.4	38.5***	15.3	43.1***	19.8	53.2***
Disability (%)						
No	70.5	43.0	64.5	30.4	62.3	36.2
Mild	18.7	25.4	23.4	32.9	24.6	34.5
Severe	10.8	31.6***	12.1	36.7***	13.1	29.3***
Number Chronic diseases (mean)	1.3	2.0***	1.6	2.3***	1.9	2.5***
COPD	10.6	25.7***	12.7	24.1***	14.3	25.4***
Heart diseases	18.2	31.6***	22.7	42.3***	25.9	46.0***
Artery disease	8.6	12.4*	11.3	36.7**	11.4	23.8**
Stroke	4.8	13.2***	6.3	16.5***	7.1	22.2***
Diabetes	6.1	21.1**	7.4	16.5**	8.5	11.5
Arthritis	35.0	32.5	46.4	39.2	51.5	57.1
Cancer	8.4	16.7**	10.8	21.5**	13.0	15.9
Difficulty Seeing (%)	21.1	29.6*	21.9	33.8*	22.6	34.4*
Difficulty Hearing (%)	31.8	38.8	34.4	40.0	31.6	42.2
Self-rated Health Fair to poor (%)	36.0	52.2***	34.7	68.4***	34.0	53.2***
Cognitive impairment: MMSE Score ≥ 23 (%)	5.9	24.8***	6.9	26.6***	5.8	30.5***
Depressive symptoms: CES-D Score ≥ 16 (%)	11.4	19.8**	11.9	27.3**	13.9	25.0**

Significance tests at each cycle: * significant at p < 0.05, ** significant at p < 0.01, *** significant at p < 0.001

### Association of end-of-life status with health care use

Table 4 shows the effect of end-of-life status on the use of care, using logistic regression models based on generalized estimating equations. In model 1, adjusting for age and gender only, end-of-life status had a significant effect on the use of all types of care except on contacts with medical specialists, which did not reach significance (p = 0.092). After adjusting for enabling variables (model 2), the regression coefficients did not change much, and for some types of care showed an increase, possibly indicating a suppressor effect of one or more of the enabling variables. Only in the case of professional home care, a small part of the effect of end-of-life status (11%) was accounted for. However, after adjusting for need variables (model 3), the coefficients showed a reduction relative to the coefficients in model 1, ranging from 82% for hospital admission to 41% for institutional care. The effect of end-of-life status on acute care and on informal personal care was no longer significant, and the effect on professional home care was only marginally significant (p = 0.089). Only the effect on institutional care was still significant (p = 0.048). Thus, the end-of-life group still tended to be more likely to use professional home care and were more likely to live in a care institution than survivors, than accounted for by the predisposing, enabling and need variables included in this analysis.

**Table 4:** The association of end-of-life status with acute and long-term care utilisation: adjusted for all predisposing, enabling and need variables (logistic regression coefficients from Generalized Estimating Equations)

	Model 1^1^	Model 2^2^	Model 3^3^
Acute care: Contact with medical specialists	0.22^+^	0.27^+^	-0.11
Acute care: Hospital care	0.77***	0.61*	0.14
Long-term care: Informal personal care	1.43***	1.59***	0.66
Long-term care: Professional home care	1.78***	1.58***	0.78^+^
Long-term care: Institutional care	1.11***	1.33***	0.65*

^1 ^adjusted for age and gender (predisposing variables)

^2 ^adjusted for age and gender (predisposing variables), education, income, urbanicity, partner status, and number of children within 15 minutes travel distance (enabling variables)

^3 ^adjusted for age and gender (predisposing variables), education, income, urbanicity, partner status, and number of children within 15 minutes travel distance (enabling variables), and functional limitations, disability, physical performance, number of chronic diseases, seeing, hearing, self-reported health, cognitive impairment, and depressive symptoms (need variables)

^+ ^significant at p <0.10, * significant at p < 0.05, ** significant at p < 0.01, *** significant at p < 0.001

Table 5 shows the independent predictive ability of all predisposing, enabling and need variables for the use of the five types of care, in addition to end-of-life status (complete model 3, Table 4). From the Wald chi-square statistic (Table 5, last line), it can be derived that the model for contacts with medical specialists had the best fit, followed by the model for hospital admission. The three models for long-term care each had a lower model fit statistic, although still highly significant.

**Table 5:** Determinants of acute and long-term care utilisation: end-of-life status and predisposing, enabling and need variables (logistic regression coefficients from Generalised Estimating Equations)

	Acute care		Long-term care		
Determinants	Medical specialist	Hospital care	Informal personal care	Professional home care	Institutional care

End of life	-0.109	0.141	0.656	0.781^+^	0.649*
					
***Predisposing variables***					
Age per year	0.017***	0.007	0.001	0.079*	0.111***
Female sex	-0.183*	-0.234*	-0.296	-0.517	-0.765*
					
***Enabling variables***					
Education (1–3)	0.147*	-0.064^+^	0.412^+^	0.567	0.397
Income (per €100)	0.002	0.02	0.006	-0.036	-0.167**
Partner	0.218**	0.210^+^	2.187***	-0.498	-0.48
Children nearby	-0.007	0.012	0.170**	0.185*	-0.04
Urbanicity (1–3)	0.198***	0.04	0.018	-0.420*	0.046
					
***Need variables***					
*Disability-related*					
Functional Limitations (0–9)	-0.02	0.008	0.259***	0.520***	0.287***
Disability (1–3)	0.350***	0.368***	0.397^+^	0.501*	-0.047
Performance (3–15)	0.02	0.060**	0.474***	0.102	0.249**
*Disease-related*					
Number Chronic Diseases	0.425***	0.151***	0.101	0.317*	-0.088
Vision	0.126*	0.042	-0.119	0.054	0.101
Hearing	0.031	-0.012	-0.157	0.08	-0.003
Self-Rated Health	0.338***	0.204**	0.092	0.124	-0.109
Cognitive impairment (0–30)	0.034*	-0.001	0.003	0.012	-0.065^+^
Depressive symptoms (0–60)	-0.004	0.007	0.015	0.029	0.009
					
Model fit (Wald chi^2 ^(df = 17))	669.85	320.05	236.91	181.05	210.14

^+ ^significant at p < 0.10, * significant at p < 0.05, ** significant at p < 0.01, *** significant at p < 0.001

Concerning predisposing characteristics, respondents with a higher age had more contacts with medical specialists and were more likely to use professional home care and institutional care, however, age was not associated with hospital admission and informal personal care. Males were more likely to have contacts with medical specialists, to be admitted to a hospital, and to live in a care institution than females. No significant gender differences were observed for informal and professional home care.

Concerning enabling variables, older people with a higher level of education were more likely to have contact with medical specialists. Those with a lower income were more likely to live in a care institution than their counterparts with higher incomes. Older people with a partner were more likely to have contacts with medical specialists, and to have informal personal care than those without a partner. Having children within 15 minutes travel distance increased the likelihood of using both informal personal and professional home care, and this variable accounted for a small part of the end-of-life effect. A higher urbanicity increased the likelihood of contacts with medical specialists but decreased the likelihood of using professional home care.

Generally, the need variables were associated with the use of any type of care, but there were notable differences in the strength of their association. The use of acute services was clearly associated with a higher number of chronic diseases, disability, vision impairment and fair to poor self-rated health, but not or not to the same extent with functional limitations, poor physical performance, hearing impairment and depressive symptoms. A higher score on the cognitive test was positively associated with contacts with medical specialists. Receiving informal personal care was associated only with the disability-related variables (functional limitations and performance), but not with the other need variables. Receiving professional home care was associated with functional limitations and disability, and in addition with the number of chronic diseases. Institutional care was association with functional limitations and physical performance, but not with the other need variables.

Running the regression models with the separate chronic diseases instead of the number of chronic diseases (data not shown), revealed that heart diseases, artery disease, diabetes, respiratory diseases, and arthritis significantly increased the probability of contacts with medical specialists. In addition, stroke was associated with receiving informal personal care, heart disease with admission to a hospital and receiving professional home care, and cancer with admission to a hospital. Institutional care was not independently associated with any of the specific diseases.

## Discussion

This study showed that those who were in their final year of life clearly used more care than those who were to survive at least three years. This was true for both acute care (contact with medical specialists and hospital admission) and long-term care (informal personal care, professional home care, and institutional care). The end-of-life effect on the use of acute care could be attributed to factors in all three categories defined by Andersen and Newman [15], but especially to need factors as was shown by the stepwise approach we used to model fitting. This indicates that older persons who die within one year more frequently use acute care than survivors, predominantly because of their more severe health problems. The greater use of long-term home care among the end-of-life group could also be largely attributed to the three categories of factors. After adjustment for these factors, the end-of-life effect on institutional care still remained significant, indicating that additional factors not included in this analysis influence the use of this type of care. A recent review showed the importance of distinguishing frailty from chronic diseases and disability for understanding health decline in older people [33]. In a previous study of our group, frailty indicators indeed had an unique effect on institutionalization of older people after taking into account chronic diseases and disability [34]. Several frailty indicators were not included in this study, such as weight loss and incontinence. Another potentially important, unexplored factor is self-management ability [35]. Factors like these might contribute to the explanation of the end-of-life effect on institutional care.

Among the factors that (partly) contributed to the end-of-life effect on utilisation of care services, we found a difference between the kind of health problems explaining the use of acute and long-term care. The end-of-life effect on long-term care was explained by disability-related variables, whereas its effect on acute care was additionally explained by the number of chronic diseases and self-perceived health. These findings confirm that utilization of long-term care is not primarily disease-related but related to the consequences of disease: impairments, disabilities and handicaps [36]. Cognitively healthy people were more likely to have contact with medical specialists as compared to those who were cognitively impaired. This finding corresponds to the findings reported by Jakobsson et al. [17] and might be explained either by a higher awareness of the services of medical specialists whom they can turn to in case of health problems, by better communication skills on the part of cognitively healthy people, or by greater reluctance of general practitioners to refer cognitively impaired patients. This finding highlights that poor cognitive ability may hamper access to appropriate care. Older people with vision impairment were also more likely to have contact with a medical specialist. Vision impairments are highly salient and disabling so that older people may seek medical help more easily for these than for other ageing-related health problems.

The enabling factors did not substantially account for the end-of-life effect on care utilization. However, the direct associations found between several enabling factors and care utilization deserve some comments. Our finding of an increased likelihood of contacts with medical specialists among the highly educated may be explained along the same lines as suggested for the cognitively healthy. In addition, low-educated older people are likely to have relatives who are also low educated, who in turn are more likely to suffer from diseases and disabilities [37]. Thus, low-educated older people may have fewer relatives who are able to care for them than higher educated peers. The finding that older adults with lower income were more likely to live in a long-term care facility than their counterparts is less easy to explain. Continuing to live in the community when needing intensive care may be more complex and costly than moving to an institution. In the Netherlands every citizen is insured for the use of both professional home care and institutional care. However, an income-dependent threshold for co-payment exists for both. It may be that the financial situation of older people with low incomes is prohibitive for the co-payment of intensive professional home care, so that they are forced to give up living in the community sooner than older people with higher incomes. Another possibility is that people with lower incomes do not prefer to continue trying to maintain themselves in the community and find institutionalization a proper alternative. On the other hand, the high co-payment required for institutional care of people with accumulated wealth may provide a high threshold to institutionalization for those in the higher income brackets. This might have changed in the years after 2002, because professional home care was moved from the Exceptional Medical Expenses Act to the Social Support Act in 2007. Municipalities that are responsible for carrying out the Social Support Act are free to decide the level of co-payment for their citizens, although there is a maximum level.

Having a partner and having a child living at less than 15 minutes travel distance were found to increase the likelihood of using informal personal care. Partners and children are an important source for providing informal care, as has been found in many previous studies [38,39]. More unexpectedly, having a partner was associated with contacts with medical specialists and having children living nearby was associated with the use of professional home care, the latter association accounting for a small part of the end-of-life effect. These associations partly corroborate findings by Bickel [9] and Jakobsson et al. [17] and suggest that partner and children facilitate the access to these care services, for example by locating appropriate care services, offering transportation, or dealing with the paper work.

A positive association was found between urbanicity and contacts with medical specialists, perhaps reflecting the better accessibility of this type of care in urban areas. The inverse association found between urbanicity and the use of professional home care may reflect a lesser availability of this type of care in densely populated areas as evidenced by longer waiting lists, but may also reflect the specific composition of the older population in urban areas. Older people in densely populated urban areas are likely to be partnerless and have low incomes [40] – factors that may hamper the access to professional home care, as suggested above.

Even after adjusting for need and enabling factors, predisposing factors (age and gender) still had some unique effects on the use of several acute and long-term care services. The effects showed a stronger association of age with institutional care, professional home care and contacts with medical specialists in the full sample. Age may therefore indicate the residual frailty-effect that we did not capture with specific frailty markers. However, in the end-of-life group, higher age was associated with less often having contacts with medical specialists and admissions to a hospital, as shown in Figure 1a and 1b. These findings are in agreement with reports by Bickel [9] and Menec et al. [11], showing an opposite pattern of hospital and long-term care utilization with age at death. Furthermore, for frail older people hospital admission may not be considered appropriate due to its likelihood of serious side-effects unrelated to diagnosis or treatment of acute illnesses, such as functional decline, depression, confusion, falls, undernutrition, and incontinence [41-43]. Instead of treatment, (palliative) long-term care will be more likely allocated to frail older-old people due to their frequent multimorbidity and decline in physical functioning [44]. This finding corresponds to health care policy in the Netherlands, where specially trained nursing home physicians see to it that nursing home residents are hospitalized as little as possible, while their quality of life is kept as high as possible.

More older men as compared to older women had contact with medical specialists and older men were more likely to be admitted to a hospital or lived in a care institution. As a first explanation for these gender effects might be proposed a greater frailty of older men in our sample. In view of the ample evidence that women live longer but in poorer health than men, however, this explanation does not seem likely. Another explanation may be that men are more effective in organizing care, that care is more easily allocated to older men, or that women are more hesitant in seeking specialist care. These gender effects require further research.

This study on the use and determinants of end-of-life care has some important advantages as compared to earlier studies [9,16,17]. First, previous studies typically were based on persons at the end of their lives only. Because this study was population-based and prospective, we could also include older people who were not at the end of their lives as a comparison group of survivors. Second, earlier studies covered only a selection of care services and had limited availability of predictor variables. Instead, we were able to include several types of both acute and long-term care and a broad spectrum of enabling and need variables which is a major advantage compared to earlier studies.

Nevertheless, some potential methodological caveats concerning this study must be addressed. First, the absolute number of older adults using care in this study is affected by stratification on end-of-life group. Thus, the numbers presented do not accurately reflect the use of care in the general older population in the Netherlands during the study period. Second, we addressed care utilization by older persons in their last year of life, which means that they could have died in any of 12 months after completing one of the cycles. The greatest increase in health care utilization takes place in the last months of life. Several studies found increases in the utilization of health services in the months before death, especially during the last month [45-47]. Thus, different percentages and explanatory models of care utilization might have been found if a shorter period before death in the end-of-life group had been used. For future research we recommend to include time from service use until death. Third, our measures of care utilization were based on self-reports, which may be inaccurate due to memory distortion. However, based on earlier research it may be expected that estimates of differences in utilization between groups of people are not affected [48]. Fourth, although we distinguished between several types of acute and long-term care, we used a global measure of these types of care: use or no use. More detailed approaches to the measurement of care utilization would involve its frequency or duration. Using different measures of care utilization may yield different results. Fifth, as Lyons and Zarit [49] noted, informal care and professional care do not exist side-by-side as separate processes. In this study, however, we did not take combinations of informal care and professional acute or long-term care into account, which are frequently present in the last year of life and are likely to enable dying at home [45]. Sixth, some types of care measured in this study might exclude each other. It is not possible for residents in long term care facilities or patients who are in the hospital at the time of measurement to be receiving home care at the same time. This means that these models may not be complete. Finally, the associations of predisposing, enabling and need variables with care utilization found in this study should be regarded as descriptive without indication of causality, as data on care utilization and its predictor variables were measured at the same time. However, our focus was on determining and explaining the differences in care utilization between older persons at the end of their lives and older persons who were to survive for at least several years.

## Conclusion

The results of this study among older people showed that need factors are decisive in the explanation of the increase in use of care services towards the end-of-life. The importance of frailty indicators in addition to chronic diseases and disability as determinants of care utilization was discussed, and this needs further research. Information on the contribution of frailty, multimorbidity and disability and combinations of these with predisposing and enabling factors contribute to understanding why older people in their last phase of life are more likely to use acute and long-term care. The insights gained from this study are essential for an effective delivery of care to the benefit of older persons themselves, their informal caregivers and professional health care providers.

## Competing interests

The authors declare that they have no competing interests.

## Authors' contributions

AP and FP participated in the data analysis and drafted the manuscript; DD conceived of and designed the study, participated in the data analysis, and helped draft the manuscript; GV, MP, MBG contributed to the interpretation of the data and commented on the manuscript. All authors approved of the final manuscript.

## Pre-publication history

The pre-publication history for this paper can be accessed here:


